# Induction of Tertiary Lymphoid Structures With Antitumor Function by a Lymph Node-Derived Stromal Cell Line

**DOI:** 10.3389/fimmu.2018.01609

**Published:** 2018-07-16

**Authors:** Genyuan Zhu, Satoshi Nemoto, Adam W. Mailloux, Patricio Perez-Villarroel, Ryosuke Nakagawa, Rana Falahat, Anders E. Berglund, James J. Mulé

**Affiliations:** ^1^Immunology Program, Moffitt Cancer Center, Tampa, FL, United States; ^2^Department of Biostatistics and Bioinformatics, Moffitt Cancer Center, Tampa, FL, United States; ^3^Cutaneous Oncology Program, Moffitt Cancer Center, Tampa, FL, United States

**Keywords:** tertiary lymphoid structures, stromal cells, lymphogenesis, tumor-infiltrating lymphocytes, immune checkpoint proteins

## Abstract

Tertiary lymphoid structures (TLSs) associate with better prognosis in certain cancer types, but their underlying formation and immunological benefit remain to be determined. We established a mouse model of TLSs to study their contribution to antitumor immunity. Because the stroma in lymph nodes (sLN) participates in architectural support, lymphogenesis, and lymphocyte recruitment, we hypothesized that TLSs can be created by sLN. We selected a sLN line with fibroblast morphology that expressed sLN surface markers and lymphoid chemokines. The subcutaneous injection of the sLN line successfully induced TLSs that attracted infiltration of host immune cell subsets. Injection of MC38 tumor lysate-pulsed dendritic cells activated TLS-residing lymphocytes to demonstrate specific cytotoxicity. The presence of TLSs suppressed MC38 tumor growth *in vivo* by improving antitumor activity of tumor-infiltrating lymphocytes with downregulated immune checkpoint proteins (PD-1 and Tim-3). Future engineering of sLN lines may allow for further enhancements of TLS functions and immune cell compositions.

## Introduction

Secondary lymphoid organs (SLOs), which are initiated in a genetically programmed process prenatally or postnatally, provide a specialized microenvironment for naïve T cell priming by antigen-presenting cells (APCs) draining from peripheral tissues (1, 2). In addition, to mediate the adaptive immune response, SLOs also participate in immune self-tolerance by maintaining recirculating Foxp3+ regulatory CD4+ T cells (Tregs) (3). To fulfill these essential immune-fate deciding functions, SLOs such as lymph nodes (LNs) require a well-organized and highly complex structure. LNs are composed of segregated T cell zones and B cell follicles, dendritic cell (DC) clusters, high endothelial venules (HEVs), and a supportive stromal reticular network (4, 5). Accumulating evidence suggests that specific immune reactions may also occur outside SLOs in organs identified as tertiary lymphoid structures (TLSs) (6, 7). TLSs, also termed ectopic lymph node-like structures, are present in sites of chronic microbial infection, chronic allograft rejection, autoimmune inflammation, and tumors in both the mouse and human (6, 8, 9).

Since the nature of TLS neogenesis is to respond to chronic inflammation, there is no specific anatomic location or developmental window for TLS induction (2, 10). In the conditions of autoimmune disease, chronic inflammation, and infection in humans, TLSs have been observed in synovial tissue, salivary glands, nervous system, thyroid gland, liver, aorta, gut, and lung (8). In human cancers, TLSs have been also detected in thyroid carcinoma, hepatocellular carcinoma, colorectal carcinoma, lung cancer, breast carcinoma, melanoma, prostate cancer, ovarian cancer, and pancreas ductal carcinoma (2, 11). Although previous studies demonstrate that TLSs may be an entry site for naïve lymphocytes and a component of humoral- and cell-mediated immunity to local inflammation, the specific functions of TLSs remain elusive, especially in cancer (1, 7, 12, 13). The existence of TLSs has been reported to be associated with favorable prognosis in certain human cancers; however, no association or a detrimental prognosis value has also been described (2). The conflict in correlations arising from these studies could be attributed to cancer types, different patient pools, various stages of disease, and diverse compositions/organizations and tumor-related locations of TLSs, which highlight a critical question in the tumor-associated TLS field: do the TLSs act like an antitumor immune-activator, a protumor immune-suppressor, or a responder to a unique tumor-inducing persistent inflammation? Therefore, mouse models of immunologically functional TLSs are desirable to further understand the function of TLSs in cancer and to potentially manipulate them to enhance immune-based therapies.

It is well recognized that neogenesis of TLSs and LNs share a similar set of molecules, i.e., lymphoid chemokines: CCL19, CCL21, and CXCL13; lymphoid factors: lymphotoxin (LT) α, LTαβ, and tumor necrosis factor superfamily (14–16). Indeed, earlier mouse models utilized numerous methods to induce TLSs in various anatomic sites, such as combining over-expression of lymphoid chemokines/factors with conditional transgenic mice (10, 16, 17), adenovirus delivery (18), or biomaterials in tissue engineering (19). The development of TLSs may also use similar cellular initiators as LNs; for example, LTβ receptor (LTβR) and podoplanin double-positive stromal lymphoid tissue organizer (LTo) cells, which can express not only lymphoid chemokines to attract hematopoietic cells but also adhesion molecules to retain these cells upon LT signaling (2, 20). Consistently, primary cells isolated from embryonic mesenteric LNs and a LTα-expressing stromal cell line established from thymus also achieved some success in the creation of TLSs in the mouse (21, 22). The potential roles of stromal cells in TLSs formation have been discussed (9, 23). LN stromal cells play a major role in mediating the interaction between APCs and lymphocytes to initiate adaptive immune responses and forming structural architecture for the homeostasis and differentiation of lymphocytes. Collectively, these findings in mouse models shed light on the molecular and cellular mechanisms that regulate TLSs formation, but direct evidence showing the potential antitumor effects of these structures remains to be elucidated.

Accumulating studies have shown that tumor-infiltrating lymphocytes (TILs) are promising prognostic markers for patient survival and response to therapy in diverse types of cancer (24, 25). Adoptive cell therapy of autologous TILs has been demonstrated to achieve objective response rate of 40–50% in the treatment of metastatic melanoma (26–30). Furthermore, blockage of immune checkpoint molecules, such as PD-1/PD-L1 and Tim-3, increased T cell infiltration and enhanced antitumor efficacy of TILs in tumor mouse models (27, 31, 32). These are consistent with findings that PD-1 and Tim-3 expression have been often detected on CD8+ TILs and identified as indicators of T cell exhaustion and dysfunction (31, 33). TLSs are considered to be an important source of TILs and closely associated with TILs in breast and ovarian cancer in human, as evidenced by that patients with both high levels of TILs and TLSs density had better disease-free survival than those with only high levels of TILs (34–36). Thus, induction of TLSs in the tumor microenvironment has the potential to increase infiltration of TILs to tumor sites and improve TILs response once there. In this study, we focused on establishing a TLS mouse model and utilizing this model to understand how TLSs can be used to manipulate the antitumor immune response and potentially enhance immunotherapy applications.

## Results

### Establishment of a LN-Derived Stromal Monoclonal Cell Line

**Figure 1:**
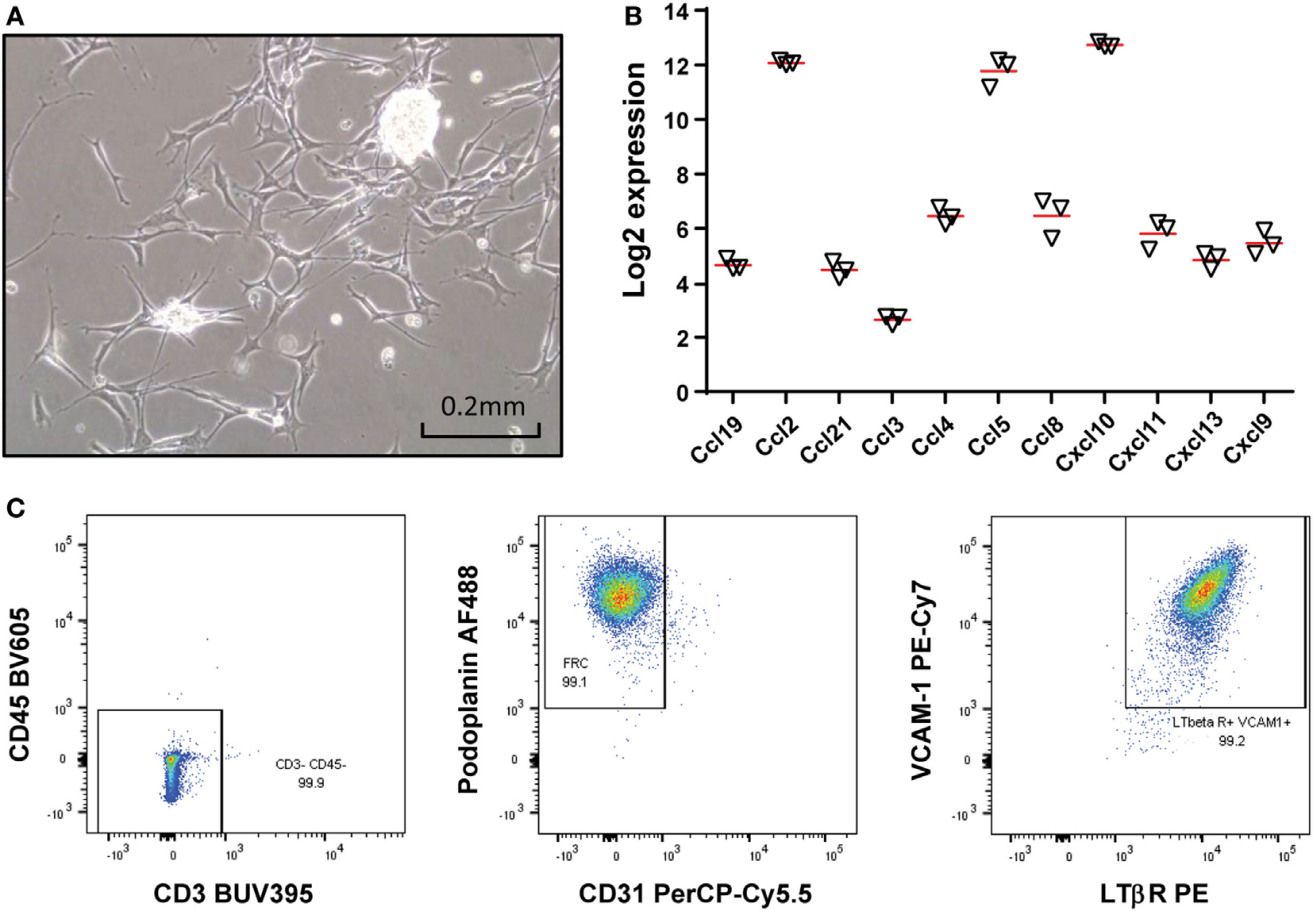
Establishing a lymph node (LN)-derived stromal cell line. **(A)** A photomicrograph of a LN-derived monoclonal stromal cell line (#2) in culture. Monoclonal cell lines were generated by limiting dilution. Scale bar denotes 0.2 mm. **(B)** Total RNA was extracted from the stromal cell line (#2) at 3 different passages and mRNA level of indicated 11 chemokines were analyzed by mouse genome arrays. Log2 transformed data were presented and red bars denote the mean. **(C)** The stromal cell line was stained for CD3, CD45, CD31, podoplanin, LTβ receptor (LTβR), and vascular cell adhesion molecule 1 (VCAM-1), and analyzed by flow cytometry. The majority of the cells are fibroblastic reticular cells with expression of VCAM-1 and LTβR.

Among eight LN-derived stromal (sLN) monoclonal cell lines that were generated, one (denoted #2 sLN) was selected and used for all experiments. #2 sLN displayed a more uniform morphology of fibroblasts compared with bulk primary sLN cells (Figure 1A; data not shown). Our previous studies showed that a chemokine gene expression signature could accurately identify the presence of tumor-localized TLSs in primary colorectal cancer (37) and metastatic melanoma (38). Therefore, expression of these chemokine genes was examined and compared between bulk primary stromal cells and #2 sLN. The #2 sLN monoclonal cell line exhibited similar to higher gene expression levels of *ccl19, ccl2, ccl21, ccl3, ccl4, ccl5, ccl8, cxcl10, cxcl11, cxcl13*, and *cxcl9* than primary stromal cells (Figure 1B; Figure S1 in Supplementary Material). Flow cytometry analysis demonstrated that #2 sLN cell line did not express CD45 or CD3, which are known lymphocyte markers (Figure 1C). The majority of the #2 sLN cells were fibroblastic reticular cells (FRCs), as evidenced by positive podoplanin and negative CD31 expression (Figure 1C). LTβR, which is a cell surface receptor for LT ligands, and vascular cell adhesion molecule 1 (VCAM-1), another adhesion marker for FRCs (4), were both expressed in the #2 cell line (Figure 1C).

### Induction of TLSs

**Figure 2:**
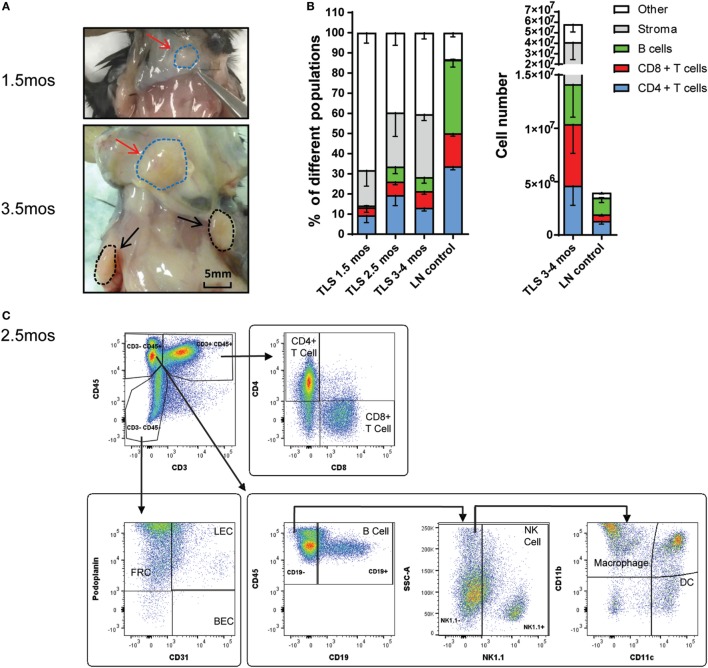
Induction of tertiary lymphoid structures (TLSs). **(A)** Representative photographs of 1.5- and 3.5-month TLSs (red arrows and blue circles) and adjacent brachial lymph nodes (LNs) (black arrows and circles). Scale bar denotes 5 mm. **(B)** Percentages and cell numbers of different cell populations in LN stroma-induced TLSs at indicated time points (*n* = 5 for 1.5 months, *n* = 3 for 2.5 months, *n* = 13–27 for 3–4 months) and LNs (control, *n* = 13–16) were analyzed by flow cytometry. Other: NK cells, macrophages, DCs, and other undefined cells. Stroma: fibroblastic reticular cells (FRCs), lymphatic endothelial cells (LECs), and blood endothelial cells (BECs). **(C)** Flow cytometry analysis of 2.5-month LN stroma-induced TLSs. Data are presented as mean ± SE.

Tertiary lymphoid structures were induced by injecting the #2 sLN cells subcutaneously in mice. Palpable structures were observed on the back of mice starting by 1.5 months (Figure 2A). The infiltration of different populations of immune cells was examined using a flow cytometry panel (Figure 2C; Figure S2A in Supplementary Material). TLSs contained 14% B, CD4+ T, and CD8+ T cells at 1.5 months, which further increased to approximately 30% at 2.5 and 3–4 months (Figure 2B). The percentages of lymphocytes in TLSs at different time points were lower, whereas the number of lymphocytes in the 3- to 4-month structures was higher than that in LNs (Figure 2B). The 2.5- to 4-month TLSs also consisted of 30% stromal cells (majority being FRCs) and 40% other cells, which included NK cells, macrophages, DCs, and unidentified cells (Figures 2B,C; Figure S2B in Supplementary Material). Furthermore, we found that there is higher percentage of activated (CD69+) and PD-1+ T cells among CD4+ and CD8+ T cells in the TLSs than that in naïve LN (Figure S2C in Supplementary Material). In addition, we observed a shift to effector memory CD4+ and CD8+ T cells (CD44+ CD62L−) in TLSs compared with naïve LNs.

### Activation of Lymphocytes in TLSs by MC38 Tumor Lysate-Pulsed DC (T-DC) Immunization

**Figure 3:**
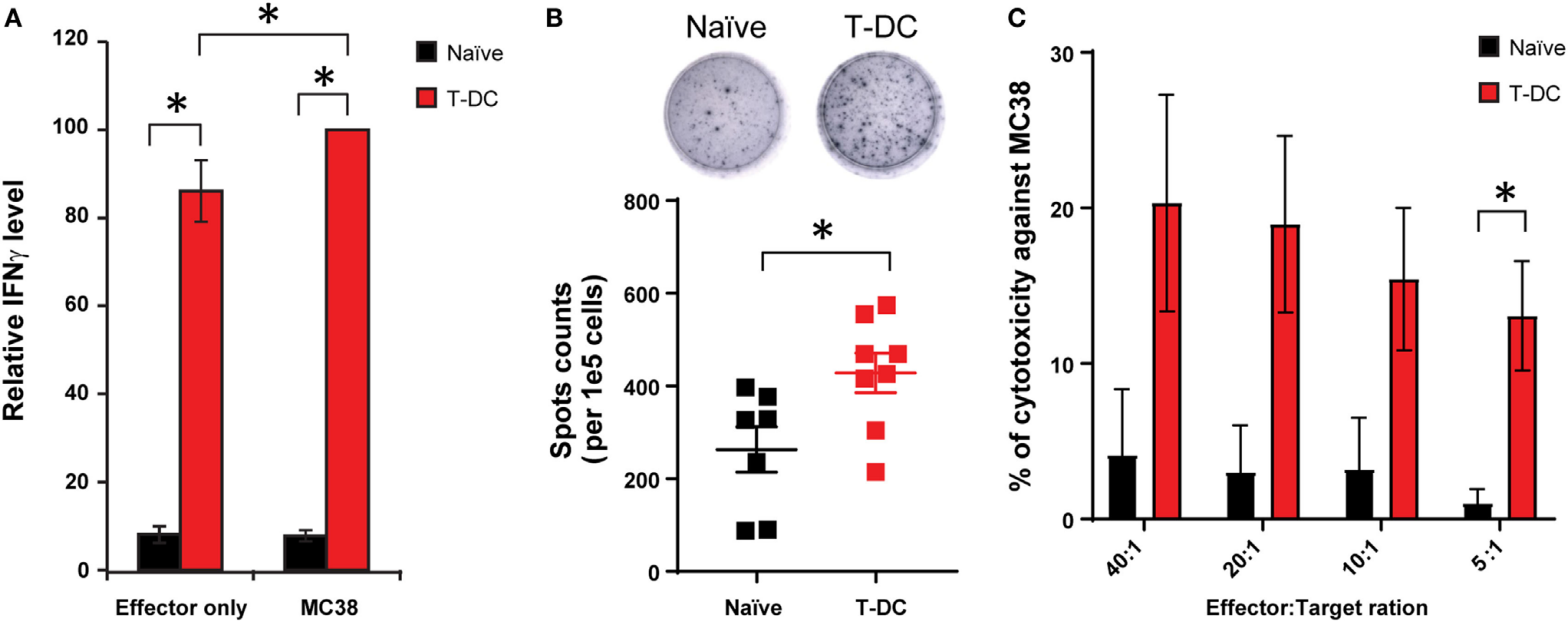
Activation of tertiary lymphoid structure (TLS)-residing lymphocytes by MC38 tumor lysate-pulsed DC (T-DC) immunization. **(A)** DCs were isolated from mouse bone marrow and pulsed with MC38 tumor lysate. 1e6 T-DCs were injected subcutaneously into TLS-bearing mice once a week for 3 weeks. T cells were isolated from the TLSs of mice immunized with T-DC vaccines or naïve mice, and incubated in medium alone (effector only group) or with irradiated MC38 cells (MC38 group) for 24 and 48 h. Supernatants were collected and tested for IFNγ levels using ELISA kits. IFNγ levels were normalized to the group of T-DC samples incubated with MC38 cells (*n* = 19–21 for naïve group, *n* = 12–15 for T-DC group). **(B)** Detection of IFNγ secretion by purified T cells from the TLSs. Representative wells were seeded with 1.25e5 cells from indicated groups. Spots were enumerated and normalized to cell number (*n* = 7 for naïve group, *n* = 8 for T-DC group). **(C)** Isolated TLS-residing T cells (effector cells) were incubated with labeled MC38 cells (target cells) at indicated ratio. Released chromium-51 was collected and measured after 5 h incubation (*n* = 3 for naïve group, *n* = 5 for T-DC group). Data are presented as mean ± SE. *p < 0.05.

In addition to confirming successful accumulations of B and T lymphocytes in the induced TLSs, we investigated whether these structures had the capacity to “educate” T cells. Bone marrow-derived DCs were pulsed with MC38 tumor lysate. The resulting T-DCs were injected into mice subcutaneously. T cells were subsequently isolated from TLSs of naïve versus T-DCs immunized mice and compared for antitumor activity by IFNγ release. T cells from TLSs of T-DC immunized mice exhibited largely enhanced baseline level of IFNγ release, which was further boosted when incubating with MC38 cells (Figure 3A). ELISPOT assay showed that the frequency of IFNγ-producing cells was significant higher in TLSs of T-DC immunized mice compared with naïve mice (Figure 3B). In addition, by chromium-51 release assay, T cells residing in TLSs of T-DC immunized mice displayed increased cytotoxicity against MC38 cells but not #2 stromal cells (Figure 3C; Figure S3 in Supplementary Material). Collectively, these findings revealed successful *in vivo* antitumor T cell priming activity within induced TLSs.

### Suppression of MC38 Tumor Growth in the Presence of TLSs

**Figure 4:**
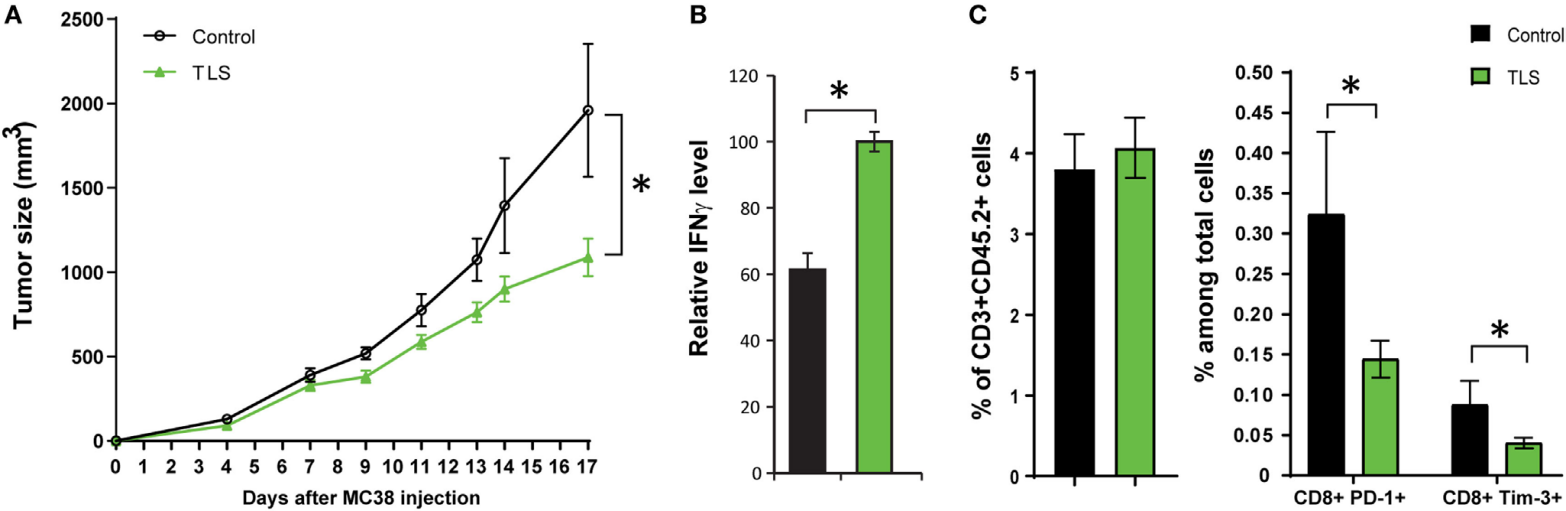
The presence of tertiary lymphoid structures (TLSs) suppresses MC38 tumor growth. **(A)** MC38 tumor growth in control and TLS-bearing mice (*n* = 3–15 for control group, *n* = 3–21 for TLS group at different time points). 1e6 MC38 tumor cells were injected subcutaneously to control and TLS-bearing mice, and tumor size was measured up to 17 days after injection. **(B)** Tumor-infiltrating lymphocytes (TILs) were isolated from MC38 tumors using CD90.2 microbeads from control and TLS-bearing mice, and incubated with irradiated MC38 cells for 24 and 48 h. Supernatant was collected and tested for IFNγ levels. IFNγ levels were normalized to the TLS group (*n* = 8 for control group, *n* = 18 for TLS group). **(C)** MC38 tumors from indicated control and TLS-bearing mice were digested and analyzed by flow cytometry. Percentage of CD3+ CD45.2+ cells (TILs) among total live cells was calculated. The expression of immune checkpoint proteins PD-1 and Tim-3 on CD8+ cells was examined from indicated groups (*n* = 8 for control group, *n* = 19 for TLS group). Data are presented as mean ± SE. *p < 0.05.

To investigate the potential antitumor function of TLSs, MC38 tumor cells were injected subcutaneously adjacent to the TLSs in C57BL/6 mice. We observed a significant (*p* < 0.05) suppression of tumor growth in TLS-bearing compared with control mice (Figure 4A). TILs were isolated and tested for IFNγ release against MC38 target cells *in vitro*. TILs from TLS-bearing mice demonstrated significantly higher IFNγ release than that in control mice (*p* < 0.05), suggesting the presence of TLSs could improve the antitumor activity of TILs in adjacent MC38 tumors (Figure 4B). We also studied TIL trafficking and composition in MC38 tumors utilizing a flow cytometry panel (39). While the infiltration of TILs was not improved in TLS-bearing mice as evidenced by similar percentage of CD3+ CD45.2+ cells, PD-1 and Tim-3 were both downregulated on CD8+ T cells in MC38 TILs in TLS-bearing mice (Figure 4C).

## Discussion

We created a mouse model of TLSs by implanting LN-derived stromal cells that express markers of FRCs. TLSs were formed by expansion of stromal cells and gradual infiltration of B cells, CD4+ and CD8+ T cells. Lymphocytes in the TLSs could be educated by T-DC immunization, and the presence of TLSs could suppress MC38 tumor growth accompanied by enhanced IFNγ release of TILs and downregulation of their expression of checkpoint inhibitors PD-1 and Tim-3. DC migration was checked using a congenic marker (transplanted DCs are isolated from CD45.1 mice, while the TLS-bearing mice are CD45.2 mice). We did not observe obvious CD45.1+ DCs migration into the TLSs, tumors, or draining LNs (data not shown). This is consistent with a previous study showing most of antigen-loaded DCs are retained at the injection site, while few live DCs reach the draining LNs and became undetectable soon after their arrival there (40). A novel mechanism for the activation of antigen-specific T cell responses upon DC vaccination has been well reviewed before (41). Antigen transfer between *ex vivo*-loaded DCs and various endogenous DC subsets is required for efficient induction of CD8+ T cells. A putative mechanism is suggested, whereby host DCs take up antigens from injected DCs that die quickly *in situ* and further prime naïve T cells in LNs. We observed abundant DCs in the TLSs, which indicates that antigen transfer between host and injected DCs could be a possible underlying mechanism of T cell induction.

The frequencies of TILs are similar between control and TLS groups, as evidenced by the similar percentage of CD3+ CD45.2+ cells (TILs) among total cells. In the presence of TLSs, there is a trend of a lower percentage of PD-1+ cells on TILs, which did not achieve significance (data not shown). Moreover, published data show that TILs isolated from MC38 tumors contain tumor-specific T cells (27). MC38 TILs when co-cultured in the presence of MC38 tumor cells had significant levels of IFNγ production compared with irrelevant tumor cells. When the same number of TILs isolated from tumors in control and TLS-bearing mice were incubated with MC38 cells, TILs in the TLS group displayed higher IFNγ release than that in the control group. Taken together, these data argue against the possibility that lower PD-1 level is due to a lower frequency of tumor-specific T cells in the TLS group. For the mechanism of PD-1 downregulation on CD8+ T cells, a previous study showed that injection of DCs engineered to express T-bet (T-box transcription factor) into murine tumors resulted in antitumor effects and rapid development of TLSs (42). Furthermore, T-bet was identified as an inhibitor of PD-1 (43). These results suggest that TLSs may downregulate PD-1 through T-bet, which warrants further investigation.

Although microarray data showed similar expression of different chemokines between the monoclonal and primary sLN cells, monoclonal sLN cells were established to induce TLS formation, due to their uniform expression of LTβR, podoplanin, and VCAM-1 that mimic LTo cells. Similarly, a previous study reported on a monoclonal sLN cell line that preserved expression of chemokine, LT pathway related receptor and lymphocyte-anchoring surface proteins as mature stroma LTo cells (44). Since stromal cells in adult LNs are considered to be direct descendants of LTo cells or their derivatives, it is likely that the adult-type cells maintain some features of embryonic organizers (4). Indeed, successful induction of “artificial” TLSs could be achieved in the renal subcapsular space with LTα-expressing monoclonal stromal cells from thymus (22). To increase the flexibility for future potential clinical practice, we induced TLSs subcutaneously. Because an earlier study revealed site-dependent differences of cytokines production between FRCs isolated from skin-draining vs. mesenteric LNs (45), we extracted sLN cells from peripheral LNs in our current work.

Comparing the cellular composition of induced TLSs to peripheral LNs, we observed: (1) lower percentage of total B and T cells; (2) disproportional ratio between B and T cells; and (3) higher percentage of stromal cells, macrophages, and NK cells. As reviewed previously, recreation of the complex architecture of lymphatic organs *ex vivo* is more challenging due to lack of proper microenvironment and efficient interaction among different cell populations, in contrast to accomplishments in the formation of human liver, blood vessels, cartilage, and skin (46). Despite relative modest size, bioengineering of LNs confronts major barriers, including the multitude of cell types, complicated and structured stromal network allowing cell motility, as well as enormous cell density on a small scale (47). The induced TLSs have a lower percentage but a higher number of lymphocytes than that in LNs, suggesting lower cell density in the TLSs will need further improvement. It is more demanding to recruit B cells than T cells, as illustrated in a study that also experienced difficulty of attracting B cells using several biocompatible materials, until the use of a sponge-like collagenous scaffold (48). Considering that a scaffold was not used in our TLSs model, the implanted sLN cells would need to proliferate to some extent to provide a 3D structure and molecule cues for host immune cell infiltration. Implantation of foreign cells and biomaterials in immune-competent animals elicits multiple cellular responses, including clearance of foreign antigens by macrophages and NK cells (49, 50). Although reports of infiltration of these innate immune cells were missing in previous TLSs mouse models, we speculate they could represent background of cell infiltration in response to a foreign substance. In addition, two recent studies showed that macrophages could play a crucial role in TLS formation, because adoptive transfer of LIGHT-stimulated macrophages could mimic intratumoral TLS induction by LIGHT (51, 52).

Tumor-associated TLSs can be positioned at/outside the tumor invasive margin (i.e., extratumoral) or within the tumor mass (i.e., intratumoral) (2). It was shown that the position of TLSs in regard to tumor could have important implications for their prognostic value in the survival of tumor bearers. For example, a recent study reported that extratumoral TLSs had a weak association with TIL frequencies in colorectal cancers derived from patients at various stages (53). Because we injected tumor cells adjacent to the structures, this design represented an extratumoral TLS model. Although we did not observe an increase of TIL number in MC38 tumors in TLS-bearing mice, we detected improved antitumor efficacy and downregulation of checkpoint inhibitory molecules. TLSs have been described as either organized lymphoid aggregates containing distinct T- and B cell zones, PNAd+ HEV, germline centers, DC-Lamp+ mature DCs, and expression of lymphoid chemokines (6) or as loose and less organized structures (2). In our current study, the induced TLSs would fall in the latter category, and preliminary multiplex immunohistochemistry results show that T cell clusters were detected, while scarce B cells did not form follicles (Figure S4A in Supplementary Material). Moreover, podoplanin+ FRCs are widely distributed with existence of CD31+ endothelium (Figure S4B in Supplementary Material). PNAd+ HEVs were also identified in the TLSs, but at a lower frequency than LNs (Figure S4C in Supplementary Material).

In conclusion, we have shown the potential of induced TLSs to mount a preventative antitumor T cell response *in vivo*. Due to the longer time to form TLSs compared with rapid MC38 tumor progression, we were unable to evaluate the impact of functional TLSs on established tumors. Our previously published studies identified important chemokines in the TLS formation. Our laboratory systematically performed microchemotaxis assays on purified immune subsets including pan-T cells, CD4+ T cells, CD8+ T cells, B cells, and NK cells, with 49 recombinant chemokines (15). We found that resting pan-T cells displayed concentration-dependent chemoattraction toward CCL19 and CCL21, and concentration-dependent chemoattraction of resting B cells was restricted to CXCL12 and CXCL13. We believe a combination of CRISPR-Cas9 genome editing and genetic modification of the LN stromal cell lines to better express the key chemokines/factors by recombinant viral vectors should provide the definitive answer (as well as, in the latter case, enhance the formation and function of the TLSs). In the future, we will focus on reducing the induction time by combining over-expression of lymphoid chemokines/factors in stromal cells with usage of proper biomaterials as a scaffold (16). Because not all human solid tumors show the presence of TLSs, the concept of constructing “designer” TLSs in “immune-cold” tumors to potentially enhance immunotherapies seems attractive.

## Materials and Methods

### Animals

Female C57BL/6 mice (6–8 weeks old) were purchased from Charles River Laboratories. Mice were housed at the Animal Research Facility of the H. Lee Moffitt Cancer Center and Research Institute. Mice were humanely euthanized by CO_2_ inhalation according to the American Veterinary Medical Association Guidelines. Mice were observed daily for specific clinical signs of discomfort and were humanely euthanized if a solitary subcutaneous tumor exceeded 2.0 cm in diameter or when mice showed signs referable to metastatic cancer. 1e6 MC38 cells were injected to control or TLS-bearing mice subcutaneously. Tumor length (*L*) and width (*W*) were measured using a clipper and tumor volumes were calculated using formula: *V* = (*L* × *W* × *W*)/2. All animal experiments were approved by the Institutional Animal Care and Use Committee and performed in accordance with the U.S. Public Health Service policy and National Research Council guidelines.

### Establishment of Monoclonal Stromal Cell Lines and Induction of Subcutaneous TLS

Stromal cells in mouse LNs were isolated as described previously (54). Briefly, peripheral skin-draining LNs in C57BL/6 mice were dissected, digested, disaggregated, and filtered into single-cell suspension, followed by incubation in complete RPMI medium (Corning Inc.). After settlement of stromal cells, medium was replaced to discard floating cells in the supernatant. Monoclonal stromal cell lines were generated at limiting dilution following a previous protocol (55). Trypsin EDTA (Corning Inc.) was used to treat the primary cells several times to remove fibroblasts with the residual attached cells growing to confluency. Then, the residual cells were diluted and aliquoted to two 96 wells with approximately 0.5 cells/well. Eight colonies were picked and expanded. The #2 sLN line, which could be passed through to at least passage 38, was selected because of shortest doubling time and used in all experiments. The doubling time of the #2 sLN line was estimated to be around 24 h. The #2 sLN cells around passage 20 were harvested from culture, washed two times with PBS, and diluted in PBS at 2e6 or 4e6/ml PBS. The #2 sLN cell suspension in 100 µl PBS was injected into each mouse subcutaneously in the middle of the back to avoid interference from endogenous LNs (brachial). The outgrowths were closely monitored and analyzed phenotypically and functionally.

### RNA Isolation and Microarray Assay

RNA was extracted from the #2 sLN line at passage 17, 18, and 19 using RNeasy Plus Mini Kit (QIAGEN). One hundred nanograms of total RNA were amplified and labeled with biotin using the Ambion Message Amp Premier RNA Amplification Kit (Thermo Fisher) following the manufacturer’s protocol initially described by Van Gelder et al. (56). Hybridization with the biotin-labeled RNA, staining, and scanning of the chips followed the prescribed procedure outlined in the Affymetrix technical manual and was previously described (57). The oligonucleotide probe arrays used were the GeneChip Mouse Genome 430 2.0 Arrays (Affymetrix), which contain over 45,000 probe sets representing over 39,000 transcripts. The arrays were normalized using IRON (58), log2 transformed, and quality controlled using sample to sample scatter plots.

### Flow Cytometry

Stromal cells were collected by trypsin and prepared by passing cells through a 40-µm cell strainer. The resulting single-cell suspensions were stained in FACS buffer with the following antibodies for flow cytometric analysis: anti-mouse CD3 (BD Bioscience), and anti-mouse CD45, CD31, Podoplanin, LTβR, VCAM-1 (All from BioLegend). TLSs were dissected from mice, mechanically dissociated and digested with tumor digestion buffer and GentleMACS (Miltenyi Biotec). After lysis of RBCs, the single-cell suspensions were analyzed by flow cytometry with the following antibodies: anti-mouse CD3, CD4, CD11b, CD11c (All from BD Bioscience), anti-mouse CD8, CD19, CD45, CD31, Podoplanin (All from BioLegend), and anti-NK1.1 (eBioscience). MC38 tumors were processed as above and stained with the following antibodies: anti-mouse CD3, CD4, CD69, CD27, CD45RA, PD-1, LAG3, and CD127 (All from BD Bioscience), and anti-mouse CD8, CD62L, CD44, KLRG1, CTLA-4, Tim-3, and CD45.2 (All from BioLegend). DAPI (Sigma-Aldrich) was used as a cell viability marker. The cells were analyzed by the LSR II flow cytometry equipped with five lasers (BD Biosciences), and the data were analyzed with Flow Jo (Tree Star).

### Tumor Lysate-Pulsed DCs

To investigate antigen-presentation and T cell priming, murine bone marrow cells were isolated from CD45.1 congenic mice and cultured for 6 days in IL-4 and GM-CSF supplemented RPMI complete medium, followed by purification of DCs using OptiPrep (Sigma-Aldrich). T-DCs were generated by incubating isolated DCs with MC38 tumor lysate at 1:3 ratio overnight. On the next day, T-DCs were collected and washed in PBS twice. 1e6 T-DCs were administrated subcutaneously in the shoulder blade area directly adjacent to the TLSs once a week for 3 weeks. One week later, T cells were isolated from TLSs for further experiments.

### Isolation of T Cells From TLSs and Tumors

Single-cell suspensions from digestion of TLSs and MC38 tumors were stained with CD90.2 microbeads following manufacturer’s protocol (Miltenyi Biotec). CD90.2-positive cells were sorted in AutoMACS (Miltenyi Biotec) and cultured in completed RPMI medium supplemented with 3,000 IU recombinant IL2 (Prometheus) for 2 h. Then, non-adherent cells were collected, counted, and seeded in 24-well plates at 2e6/well. On the next day, the isolated T cells were used in different functional assays, as described below.

### ELISA and ELISPOT

For detection of IFNγ release, T cells isolated from TLSs and MC38 tumors were mixed with irradiated MC38 cells at a ratio of 10:1 or not in 96-well plates. Culture supernatants were collected after 24 and 48 h, and IFNγ production was measured with an IFNγ ELISA kit (BD Bioscience). Isolated T cells were seeded at 1.25e5/well, and the number of IFNγ-producing cells was measured using a mouse IFNγ ELISpot Kit (R&D systems). The number of positive spots was enumerated using an automatic ELISPOT counter (AID).

### Chromium Release Assay

A ^51^Cr release assay was performed as described previously (27). MC38 cells were used as targets. TLS-residing T cells were extracted and used as effector cells. Briefly, MC38 cells were labeled for radioactivity with 100 μCi of ^51^Cr (Amersham Corp.) for 2 h at 37°C in a CO_2_ incubator. The labeled cells were washed with HBSS and added to the effector cells in at least triplicate wells of 96-well round-bottomed microplates with effector to target ratio at an initial 40:1 and subsequent 1:2 dilutions until 0.15:1. Labeled target cells only were used as minimum release, while target cells lysed by TritonX-100 were used as maximum release. After 5 h, supernatant was harvested and measured in Trilux (PerkinElmer). The percentage of specific ^51^Cr release was determined by the following equation: (experimental release − minimum release)/(maximum release − minimum release) × 100.

### Statistical Analysis

The data were analyzed with a two-tailed Student’s *t*-test or Wilcoxon matched-pairs signed rank test by GraphPad Prism. A *p* value of <0.05 was considered statistically significant.

## Ethics Statement

All animal experiments were approved by the Institutional Animal Care and Use Committee and performed in accordance with the U.S. Public Health Service policy and National Research Council guidelines.

## Author Contributions

JM, AM, and GZ conceived and designed the experiments. GZ, SN, PP-V, and RN performed the experiments with support from AM and RF. GZ and AB analyzed the data. GZ and JM wrote the paper with input from AB and RF.

## Conflict of Interest Statement

The authors declare that the research was conducted in the absence of any commercial or financial relationships that could be construed as a potential conflict of interest.
