# Supplementary material for: Induction of Tertiary Lymphoid Structures With Antitumor Function by a Lymph Node-Derived Stromal Cell Line
Source: Front Immunol. 2018 Jul 16;9:1609. doi: 10.3389/fimmu.2018.01609 (PMC6054958; doi:10.3389/fimmu.2018.01609)
Supplement: Supplementary file 1 [file data_sheet_1.PDF]

Supplementary Material

Induction of Tertiary Lymphoid Structures with Anti-tumor Function by a Lymph Node-Derived Stromal Cell Line

Genyuan Zhu[1], Satoshi Nemoto[1,@], Adam W. Mailloux[1], Patricio Perez-Villarroel[1], Ryosuke Nakagawa[1], Rana Falahat[1], Anders E. Berglund[3], and James J. Mulé[1,2,*]

[1]Immunology and [2]Cutaneous Oncology Programs and [3]Department of Biostatistics and Bioinformatics, Moffitt Cancer Center, Tampa, FL, USA

*** Correspondence:** James J. Mulé: James.Mule@moffitt.org

Current address:

[@] Tokyo Woman's Medical University Hospital, Institute of Gastroenterology Surgery, 8-1 Kawada-cho, Shinjuku-ku, Tokyo, Japan

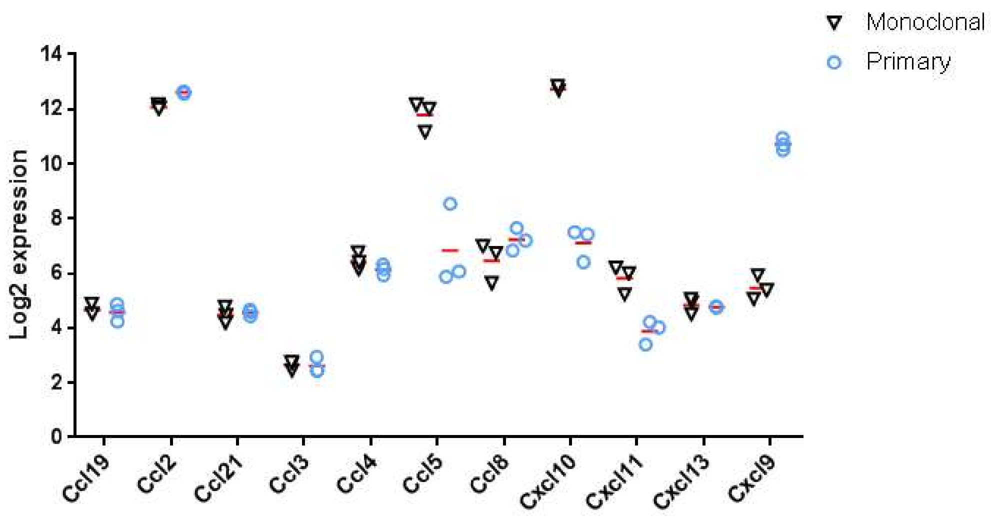

Supplementary Figure 1. Total RNA was extracted from the monoclonal or primary LN stromal cells at 3 different passages and mRNA levels of the indicated 11 chemokines were analyzed by mouse genome arrays. Primary stromal cells were isolated from peripheral LNs and cultured *in vitro*. Log2 transformed data were presented and red bars denote the mean.

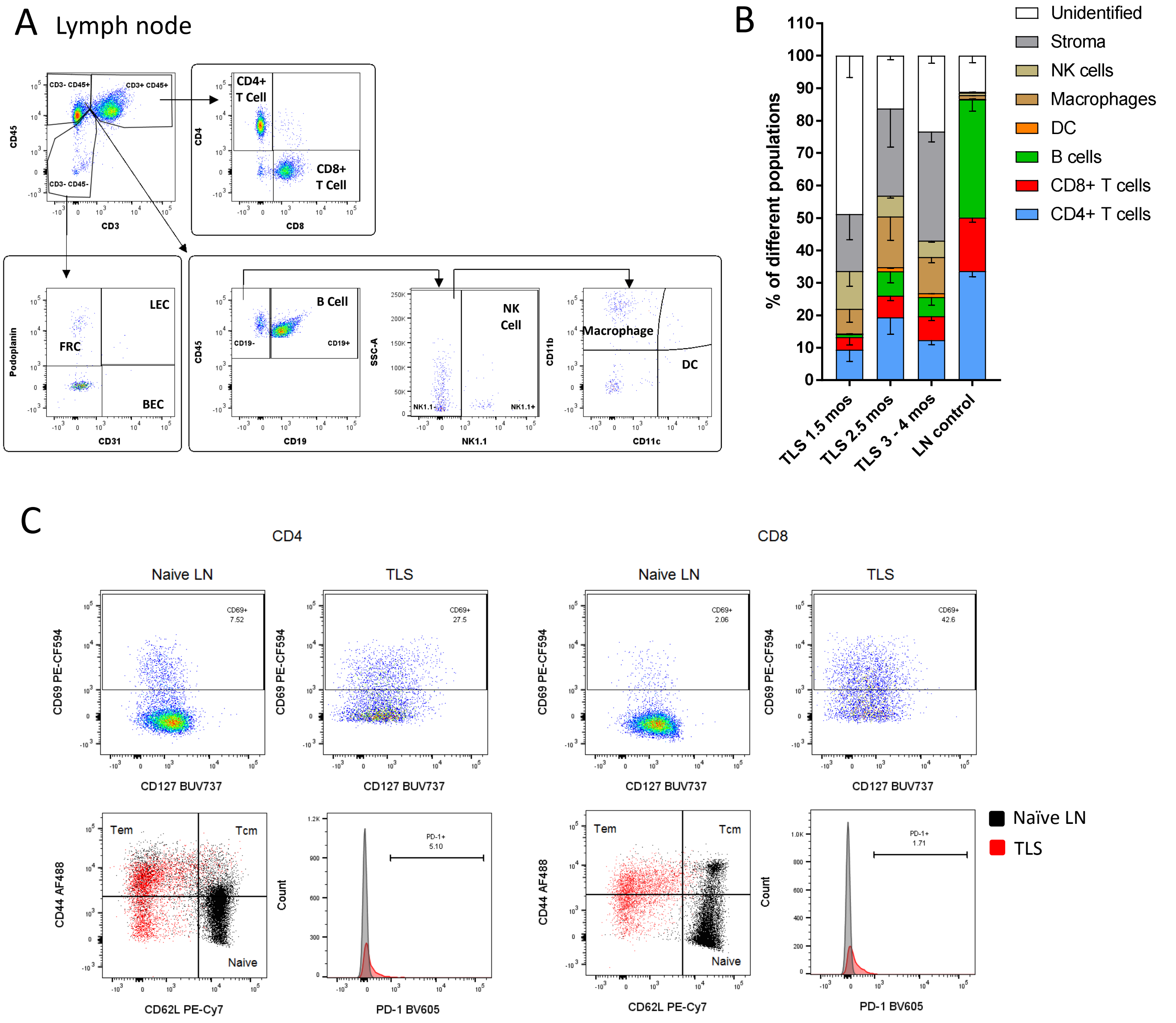

Supplementary Figure 2. (A) Representative flow cytometry analysis of LNs. (B) Percentages of different cell populations in LN stroma-induced TLSs at indicated time-points (n=5 for 1.5 months, n=3 for 2.5 months, n=27 for 3-4 months) and LNs (control, n=16) were analyzed by flow cytometry. Stroma: FRCs (fibroblastic reticular cells), LECs (lymphatic endothelial cells), and BECs (blood endothelial cells). (C) Representative flow cytometry analysis of activated (CD69+) T cells, memory phenotype (Tem: Effector memory T cells; Tcm: Central memory T cells), and percentage of PD-1 among CD4+ and CD8+ T cells in naïve LN (n=2) and 3-7.5mos TLSs (n=6).

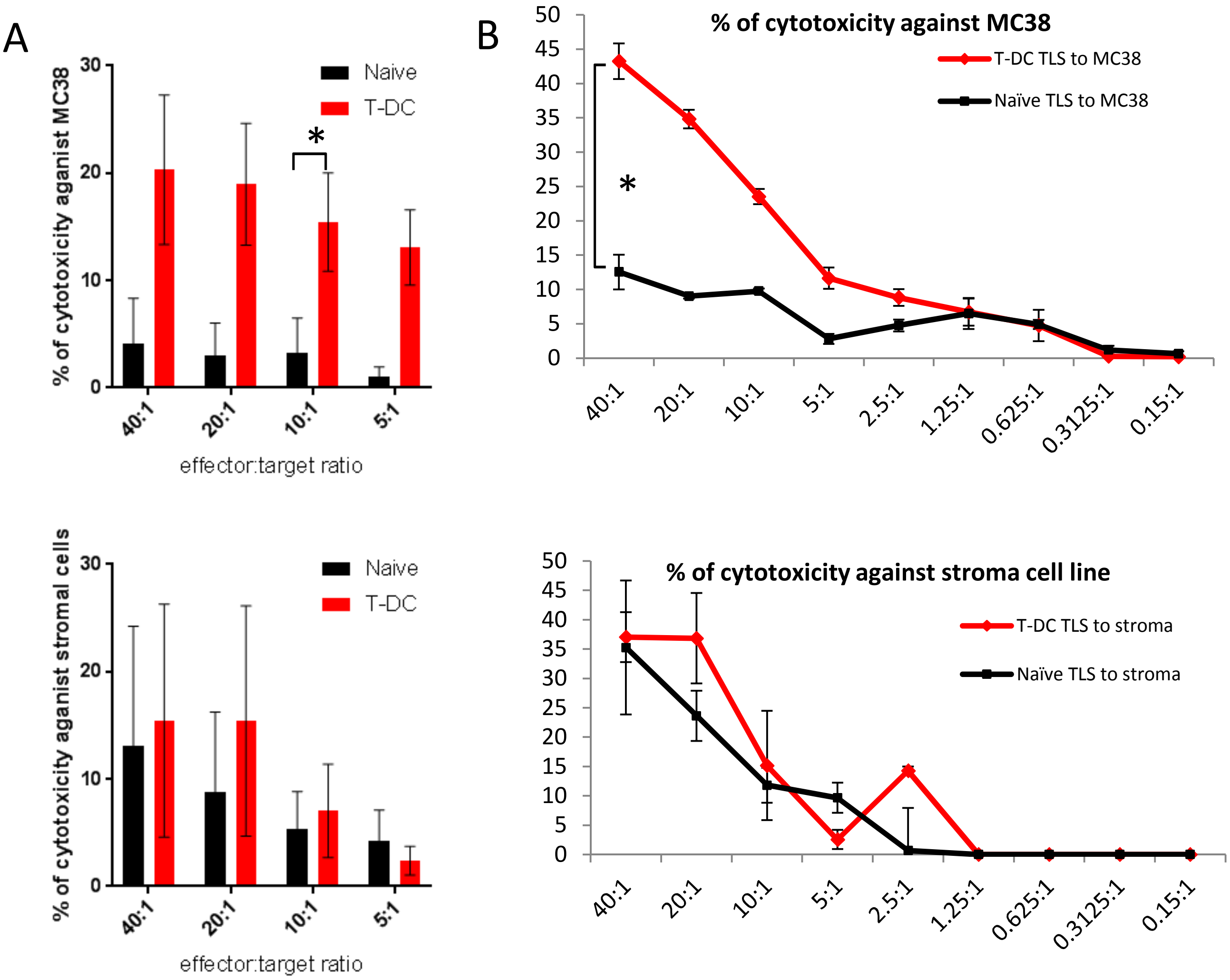

Supplementary Figure 3. (A) DCs were isolated from mouse bone marrow and pulsed with MC38 tumor lysate (T-DCs). T-DCs were injected subcutaneously into TLS-bearing mice once a week for 3 weeks. Isolated TLS-residing T cells (effector cells) were incubated with labeled MC38 or #2 stromal cells (i.e. target cells) at indicated ratios. Released chromium-51 was collected and measured after 5 hours incubation (n=3 for naïve group, n=3-5 for T-DC group). (B) Representative cytotoxicity curves for T cells isolated from naive or T-DC TLSs targeted against MC38 or #2 stromal cells. Data are presented as mean±SE. * $p<0.05$.

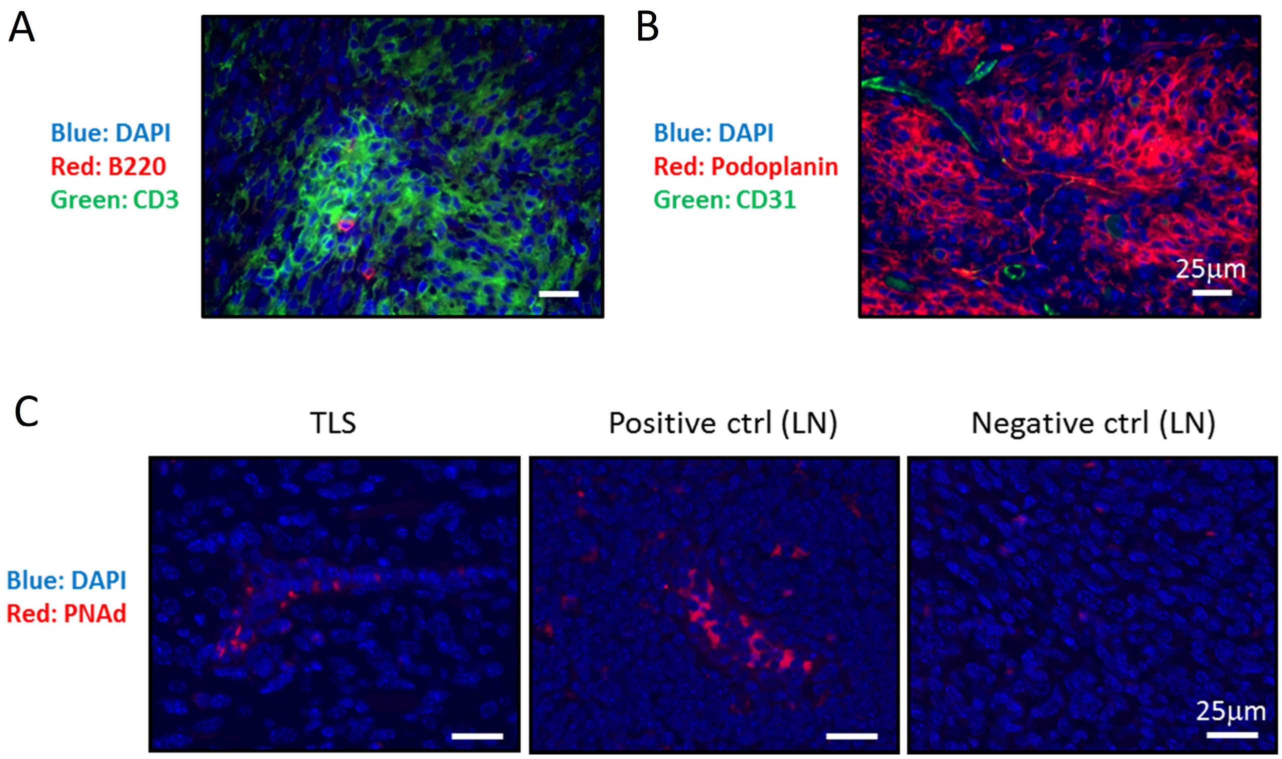

Supplementary Figure 4. (A) Double immunofluorescence staining for CD3+ T cells (green) and B220+ B cells (red) in 2.5-3.5mos TLSs. (B) Double immunofluorescence staining for CD31+ endothelial cells (green) and podoplanin+ FRCs (red) in 2.5-3.5mos TLSs. (C) Immunofluorescence staining for PNAd+ HEV (red) in 2.5-3.5mos TLSs. LN sections were used as positive and negative controls. Nuclei were stained with DAPI (blue). (Scale bar, 25 μm)

Supplementary materials and methods:

Collected samples were fixed in 10% zinc formalin (Sigma-Aldrich) for tissue analysis. Paraffin-embedded tissues were sectioned at 5 µm. Single and double immunofluorescence (IF) staining of the paraffin-embedded tissue sections were performed according to standard protocols. Tissue sections were incubated at 4 °C overnight with primary antibodies. For IF staining, the following antibodies were used: CD3e (1:100, BD Pharmingen #553058), B220 (1:100, eBioscience #14-0452-81), CD31 (1:50, Santa Cruz #sc-1506), Podoplanin (1:100, R&D Systems #AF3244-SP), and PNAd (1:50, Biolegend #120801). Fluorescence was visualized using a Leica TCS SP5 AOBS laser scanning confocal microscope or Zeiss upright fluorescent microscope, and the images were analyzed with Adobe Photoshop CC 2017.
